# The Hangman’s Tourniquet: A Safe and Practical Approach for Reducing Blood Loss During Uterine Myomectomy

**DOI:** 10.7759/cureus.50662

**Published:** 2023-12-17

**Authors:** Vishal Bahall, Lance De Barry, Keevan Singh

**Affiliations:** 1 Obstetrics and Gynaecology, The University of the West Indies, St. Augustine, TTO; 2 Obstetrics and Gynaecology, San Fernando General Hospital, San Fernando, TTO; 3 Anaesthesia and Intensive Care Unit, Department of Clinical Surgical Sciences, The University of the West Indies, San Fernando, TTO

**Keywords:** gynaecology, fibroid, fertility-sparing surgery, uterine leiomyoma, myomectomy

## Abstract

Study objectives: The application of a pericervical uterine artery tourniquet is a useful method of reducing intraoperative haemorrhage during abdominal myomectomy. However, the utilization of a single combined pericervical uterine artery and infundibulopelvic ligament tourniquet is a more effective and influential method of temporarily occluding the uterine vasculature to decrease intraoperative blood loss, reducing the requirement for blood products, the risk of conversion to hysterectomy, and patient morbidity and mortality during fertility-sparing leiomyoma surgery. Our objective of this retrospective review was to assess the effectiveness of the combined uterine artery and infundibulopelvic ligament tourniquet, which we coin as the “Hangman’s uterine tourniquet”, in reducing intraoperative blood loss during abdominal myomectomy, thereby reducing the need for blood products.

Methods: This retrospective study included 39 patients diagnosed with symptomatic subserosal, intramural, and/or submucosal uterine leiomyoma (>3 cm) who underwent an abdominal myomectomy between January 2021 and December 2022.

Results: Thirty-nine patients met the eligibility criteria for our study. The average patient age included in our study was 36 years. All myomectomies were completed with a mean intraoperative blood loss of 252.60 ml and the average number of fibroids removed was seven. The largest fibroid removed measured 27 x 20 cm in diameter while the most significant number of fibroids removed was 41. Moreover, the mean duration of the tourniquet application was 45.31 minutes, and the mean duration of operation was 80.44 minutes. Thirty-six patients (92.3%) had an estimated blood loss <500 ml. The number of fibroids removed was a statistically significant risk factor influencing estimated blood loss (p = 0.019). However, there was no statistical significance between estimated blood loss and the size of the largest fibroid removed (p = 0.178) nor estimated blood loss and a history of previous surgery (p = 0.412). The postoperative blood transfusion rate was 2.5% and no patients suffered grade III or higher surgical complications according to the Clavien-Dindo classification.

Conclusion: This study showed that the utilization of a temporary intraoperative combined pericervical uterine artery and infundibulopelvic ligament tourniquet is an effective, practical, and economical approach to limiting intraoperative blood loss during abdominal myomectomy.

## Introduction

Uterine leiomyomas are the most common tumours of the female genital tract [1]. An estimated 80% of women in the reproductive age group in the Caribbean have uterine leiomyomas and approximately one-third of these women are symptomatic and require treatment [1]. Uterine leiomyomas may be amenable to medical treatment; however, in many cases, patients require surgical intervention in the form of uterine myomectomy, uterine artery embolization, or hysterectomy [2]. Uterine myomectomy is the procedure of choice for women wishing to retain reproductive potential and/or their uterus [2].

Unfortunately, because of the highly vascular nature of uterine leiomyoma, myomectomy is associated with significant intraoperative haemorrhage regardless of the surgical approach [3]. This often leads to an increased requirement for blood or blood products, a greater risk of conversion to hysterectomy, and significant patient morbidity and mortality rates [3]. The massive blood loss associated with the dissection of large or multiple uterine leiomyomas renders uterine myomectomy a more technically challenging procedure than hysterectomy. According to Kongnyuy et al., myomectomy is associated with an estimated blood loss of 150-1,050 ml and a blood transfusion rate of 20% [4]. According to epidemiological data, the risk of conversion to emergency hysterectomy to combat severe intraoperative blood loss (>2 litres) during abdominal myomectomy is approximately 1-4% [5]. Furthermore, in resource-limited clinical settings where there are shortages in the availability of blood and blood products, myomectomy leads to greater morbidity and mortality rates, a longer duration of hospitalization, and significantly inflated patient and institutional costs.

The application of a temporary pericervical uterine artery tourniquet is one method of effectively reducing intraoperative blood loss [6]. However, the extensive network of collateral vessels that also arise from the ovarian vessels often hegemonizes and overwhelms a tourniquet applied solely to the uterine vessels. This may lead to significant blood loss during leiomyoma enucleation and the requirement of additional haemostatic measures such as intramyometrial vasopressin or intravenous tranexamic acid [6]. In this regard, the application of a single combined uterine artery and an infundibulopelvic ligament tourniquet is a more effective and practical method of safely reducing intraoperative haemorrhage during uterine myomectomy without compromising ovarian function [7].

The aim of this study was to evaluate the effectiveness of a temporary combined intraoperative uterine artery and infundibulopelvic ligament tourniquet in reducing blood loss during abdominal myomectomy.

## Materials and methods

This retrospective study was approved by The University of the West Indies Campus Research Ethics Committee (approval number: CREC-SA.2336/10/2023). All procedures performed in the study were in accordance with the ethical standards of the medical institution and research committee and with the 1964 Helsinki Declaration and its later amendments or comparable ethical standards. Prior to undergoing surgery, written informed consent was obtained from all the participants.

Surgical technique

**Figure 1:**
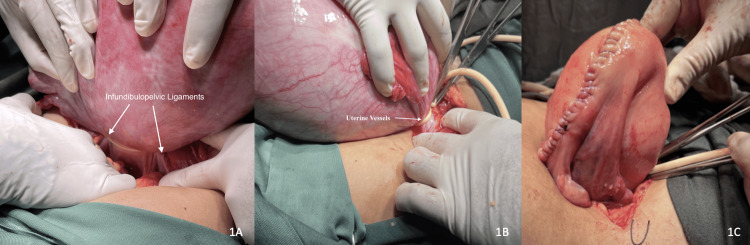
Application of single combined bilateral uterine artery and ovarian artery uterine tourniquet (Hangman’s uterine tourniquet). (A) A tourniquet looped around the bilateral infundibulopelvic ligaments. (B) The tourniquet is secured at the level of the internal cervical os and incorporates bilateral uterine vessels. (C) Uterine defects were repaired following fibroid enucleation.

In all patients, laparotomy was performed either by Pfannenstiel or midline incision and a transabdominal plane block or rectus sheath block was performed prior to abdominal entry. The uterus was exteriorized and examined for subserosal and intramural leiomyomas and other gross pathology. The adnexa were gently handled and elevated and an 18-Fr 20 cm long silicone Foley catheter was looped around both infundibulopelvic ligaments (Figure 1A). The two ends of the catheter were secured at the level of the cervix with two single-tied knots secured by two Kocher clamps so that a pericervical ring was formed (Figure 1B). The uterine tourniquet effectively incorporated both infundibulopelvic ligaments as well as both uterine vessels. The uterine tourniquet was applied for a maximum continuous duration of 60 minutes after which it was released for five to 10 minutes and reapplied if required.

Once the tourniquet was successfully applied, an incision was made using the monopolar electrode or scalpel at the area of the maximal projection of the leiomyoma from the uterine surface. The leiomyoma was then enucleated by bluntly dissecting the layers of uterine tissue surrounding it. Care was taken to ensure the safe handling of the adnexa while minimizing the number of serosal incisions to the uterus required to remove the leiomyomas. In select patients, guided by preoperative imaging and intraoperative findings, the endometrial cavity was breached, and submucosal leiomyomas were removed if present. After leiomyoma enucleation, the endometrial cavity was closed with 2-0 Vicryl® suture (polyglactin 910, Ethicon, Raritan, NJ). The myometrium was closed in layers using 1-Vicryl® suture (polyglactin 910, Ethicon, Raritan, NJ) and the uterine serosa was closed with a 2-0 polydioxanone (PDS) suture (Ethicon, Raritan, NJ) (Figure 1C).

Following the repair of all the uterine defects, the uterine tourniquet was removed and warm packs were applied to the uterus to facilitate reperfusion. Blood loss was estimated by measuring the volume of blood collected by the suction and visual estimation of swabs. All patients were discharged in satisfactory condition one to two days postoperatively and were placed on antibiotic prophylaxis and analgesics.

Statistical analysis

The data were analysed utilizing SPSS (IBM Corp., Armonk, NY). Summary statistics are given as mean + standard deviation and percentage. The independent samples t-test was applied for continuous variables. The Mann-Whitney U test was used for inter-group comparisons of parameters with a non-normal distribution. In all analyses, a value of p < 0.05 was considered statistically significant.

## Results

Thirty-nine patients were recruited from a cohort of 40 patients referred for the management of symptomatic uterine fibroids with a desire to preserve fertility. The patient demographics are summarized in Table 1.

**Table 1:** Patient demographics.

Patient demographics	Mean	Standard deviation
Age (years)	36.9	+/- 6.11
Body mass index (kg/m^2^)	27.32	+/- 3.32
Uterine size (weeks’ gestation)	18	+/- 16.86
Number of fibroids removed	6.55	+/- 9.48

**Figure 2:**
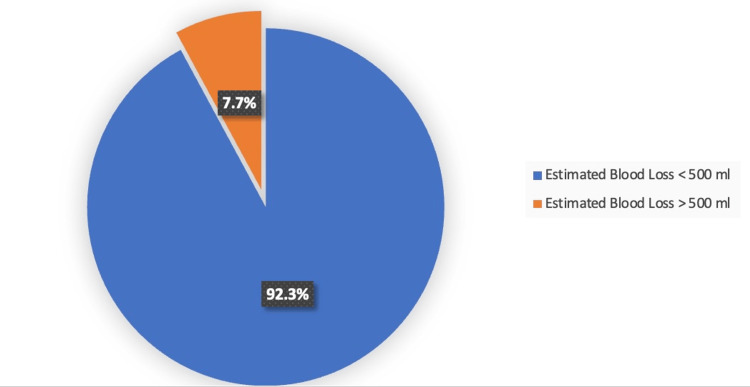
Estimated blood loss (ml) utilizing Hangman’s uterine tourniquet.

All myomectomies were successfully completed with a mean intraoperative blood loss of 252.63 ml (Table 2). Thirty-six (92.31%) patients had an estimated blood loss of less than 500 ml, while 7.69% had an estimated blood loss greater than 500 ml (Figure 2). The mean duration of the tourniquet application was 45.31 minutes, and the mean duration of operation was 80.44 minutes. The mean number of fibroids removed was 6.55, and the largest fibroid removed was 27 x 20 cm in diameter. In only three cases (7.69%), the use of additional haemostatic agents was required, with Surgicel^TM^ Nu-Knit Absorbable Haemostat (Ethicon, Raritan, NJ) used in one case and two cases requiring the use of intramyometrial adrenaline. Patients were discharged in satisfactory condition on average 26.89 hours after surgery. Only one patient remained hospitalized for 96 hours due to persistent nausea and vomiting after surgery.

**Table 2:** Intraoperative details utilizing Hangman’s uterine tourniquet. N/A - not applicable.

Intraoperative variables	Mean	Standard deviation
Number of fibroids removed	6.55	+/- 9.48
The size of the largest fibroid removed	27 x 20 cm	N/A
Estimated blood loss (millilitres)	252.60	+/- 251.46
Duration of tourniquet application (minutes)	45.31	+/- 16.86
Duration of operation (minutes)	80.44	+/- 30.73
Duration of hospitalization (hours)	26.89	+/- 13.70

The perioperative details are summarized in Table 3. The blood transfusion rate was 2.5%. No patients experienced grade III or higher surgical complications according to the Clavien-Dindo classification and 100% of patients restarted menses within six weeks after myomectomy. Postoperative haemoglobin values were not performed unless clinically indicated. In one patient with an estimated uterine size of 36 weeks, 41 uterine fibroids were removed with an estimated blood loss of 800 ml and duration of operation of 142 minutes. In this patient, the uterine tourniquet was released for approximately five to 10 minutes and reapplied after 60 minutes elapsed and intravenous tranexamic acid was utilized as an adjunct for haemostatic control. The number of uterine fibroids removed was a statistically significant factor influencing estimated blood loss (p = 0.019). However, there was no statistical significance between estimated blood loss and the size of the largest fibroid removed (p = 0.178) nor estimated blood loss and a history of previous surgery (p = 0.412).

**Table 3:** Perioperative details utilizing Hangman’s uterine tourniquet.

Perioperative details	Incidence
Postoperative blood transfusion rate	2.50%
Requirement for additional measure of haemostasis	7.69%
Conversion to hysterectomy	0%
Grade III or higher surgical complications (Clavien-Dindo classification)	0%
Ovarian function (% of patients restarting menses by six weeks post-myomectomy)	100%

## Discussion

The incidence of uterine fibroids in the Caribbean is approximately 80% and up to one-third of these women are symptomatic and require treatment [1]. Uterine fibroids may be amenable to medical treatment that includes combined oral contraceptives, cyclical progestins, levonorgestrel intrauterine systems, gonadotropin-releasing hormone (GnRH) agonists, or selective estrogen receptor modulators [8]. However, in many cases, uterine fibroids are refractory to medical therapy and may require surgical intervention in the form of myomectomy, uterine artery embolization, and hysterectomy [2]. Uterine myomectomy is the most common fertility-sparing fibroid surgery, improving both spontaneous pregnancies and in vitro fertilization (IVF) outcomes and providing symptomatic relief of uterine fibroids [9]. According to a population-based study by Yuk et al., the incidence of myomectomy is approximately 14.6 per 10,000 patients [10]. In our study, all patients were diagnosed with symptomatic uterine fibroids after clinical assessment and ultrasound imaging and then subsequently underwent abdominal myomectomy.

This retrospective study included 39 patients with symptomatic uterine fibroids of >3 cm in diameter and a histopathological diagnosis of uterine fibroids who underwent abdominal myomectomy. In our study, all myomectomies were performed utilizing a single combined bilateral uterine artery and ovarian artery tourniquet applied at the level of the internal cervical os. We have coined the term “Hangman’s Uterine Tourniquet” to describe the method of this tourniquet. Our study demonstrated a mean blood loss of 252.63 ml and a blood transfusion rate of 2.5% with the utilization of the Hangman’s uterine tourniquet. An analysis of estimated blood loss revealed a mean blood loss of less than 500 ml in 12 patients with fibroid diameters between 3 and 4 cm. Eighteen patients with fibroid diameters between 5 and 10 cm had a mean blood loss of less than 500 ml, while six patients had a mean blood loss of less than 500 ml for fibroids >10 cm in diameter. Three patients had an average blood loss of greater than 500 ml despite the utilization of the Hangman’s uterine tourniquet. In those three cases, the uterine size exceeded 20 weeks, and the number of fibroids removed was 10, 32, and 41, respectively. One limitation of this study is its clearly retrospective design.

The data demonstrated that the average tourniquet time was 45.31 minutes, and the average duration of operation was 80.44 minutes. The average number of uterine fibroids removed was 6.55, and the greatest number of fibroids removed during a single myomectomy was 41. The largest fibroid removed measured 27 x 20 cm in diameter. Patients were discharged in satisfactory condition approximately 26.89 hours after surgery. None of the patients included in this study suffered grade 3 or higher surgical complications according to the Clavien-Dindo classification. The Clavien-Dindo classification is a reporting tool that defines a complication as “any deviation from the expected postoperative course” and assigns a severity category from I-V according to the level of resources utilized to treat the complication [11]. In only three cases, the use of additional haemostatic agents was required with Surgicel^TM^ Nu-Knit Absorbable Haemostat used in one case and two cases requiring the use of intramyometrial adrenaline.

Open myomectomy is a major surgical procedure and is associated with considerable morbidity, in particular, blood loss [12]. Previous studies reported a mean blood loss in myomectomy of 150-1050 ml and a blood transfusion rate of 20% [4]. The aim of the myomectomy procedure includes removing the uterine fibroid without excessive blood loss, closing the dead space to prevent hematoma formation, ensuring a quality uterine scar that can withstand future pregnancy, and minimizing postoperative adhesion formation [2]. Several non-surgical methods have been traditionally employed to reduce blood loss during myomectomy, including the use of vasoactive drugs like vasopressin or adrenaline, tranexamic acid, uterine tourniquet, and preoperative treatment with GnRH agonists [4].

There is evidence to suggest that temporary haemostatic occlusion of the uterine blood supply is one of the most effective methods to reduce intraoperative haemorrhage during myomectomy [7]. According to a randomized double-blind trial by Mehdizadehkashi et al., uterine tourniquet application reduced intraoperative haemorrhage and shortened the duration of operation compared to cases where no uterine tourniquet was utilized [13]. Alptekin et al. also reported a significant reduction in blood loss when a uterine tourniquet was applied versus in patients in whom a tourniquet was not applied (286.4 + 137.5 ml versus 673.8 + 172.3 ml) [14]. Taylor et al. were the first to apply triple tourniquets at the level of the uterine cervix that incorporated the uterine vessels, ovary proprium, and infundibulopelvic ligaments [7]. Similar to our results, they reported reduced blood loss and reduced blood transfusion rates with no adverse effects on uterine perfusion or ovarian function.

Reassuringly, the Hangman’s uterine tourniquet did not appear to affect ovarian function as 100% of our patients restarted menses within six weeks of myomectomy. Taylor et al. also reported no change in ovarian function following the triple tourniquet technique as postoperative serum follicular-stimulating hormone (FSH) levels did not significantly change compared with preoperative levels [7]. Another study by Al et al. that focused on the effects of triple and single uterine tourniquets concluded that tourniquet use had no significant effect on ovarian reserve as determined by anti-Mullerian hormone levels [15]. The patients included in our study did not undergo FSH or anti-Mullerian hormone testing as a measure of ovarian reserve. However, all patients underwent transvaginal ultrasound at either six weeks or three months postoperatively and all patients reported menses at follow-up appointments.

The Hangman’s uterine tourniquet is a safe, practical, and cost-effective method that can be employed to perform abdominal myomectomy in clinical settings where there is a shortage of blood and blood products [16]. In our clinical setting, where the incidence of uterine fibroids is approximately 80% and there is an increasing population of women desirous of fertility over the age of 35, a significant number of patients each year undergo open myomectomy. In patients with multiple uterine fibroids, our data have shown that a myomectomy can be safely performed utilizing Hangman’s uterine tourniquet.

Limitations

Our study is a retrospective review, which typically can create selection bias and affect conclusions drawn from the data. We aimed to improve on this by including all myomectomies performed during the period January 2021 to December 2022 that met the inclusion criteria. Due to the small sample size and nature of the study being on a surgical technique, we aimed to limit the effect of confounding variables by using all the procedures done by the same surgeon and a single institution.

Our small sample size also limits the ability to make generalizations about the population. However, at present, there are limited published data on our population utilizing this combined uterine artery and infundibulopelvic ligament tourniquet to reduce intraoperative blood loss. Evidence from the literature review on techniques at myomectomy aims to reduce the limitations that this small sample size may pose. Conducting a prospective review over a longer period of time will increase sample size and reduce bias.

## Conclusions

In conclusion, this study showed that the use of a temporary intraoperative pericervical tourniquet that incorporates the uterine vessels and infundibulopelvic ligament may decrease intraoperative blood loss and reduce the need for blood transfusion during abdominal myomectomy. In our clinical setting, the procedure for abdominal myomectomy is not standardized and the techniques for reducing intraoperative haemorrhage involve the use of vasoactive substances such as vasopressin or adrenaline and uterine artery tourniquet. This novel technique, the Hangman’s uterine tourniquet that incorporates the uterine vessels and infundibulopelvic ligament, is a safe, effective, and economical method to successfully achieve a myomectomy with no need for routine crossmatching in a setting with limited availability of blood products. Moreover, the uterine tourniquet helps to shorten the duration of operation and hospitalization and ultimately increases the likelihood of spontaneous pregnancies and IVF outcomes.
